# Effects of TNF-α on penile structure alteration in rats with hyperprolactinemia

**DOI:** 10.1371/journal.pone.0181952

**Published:** 2017-08-01

**Authors:** Zhong-Lin Wang, Ling-Yu Yang, Hong-Huan Chen, Hsiao-Hsin Lin, Yi-Ting Tsai, William J. Huang

**Affiliations:** 1 Department of Physiology, School of Medicine, National Yang-Ming University, Taipei, Taiwan; 2 Department of Pediatrics, School of Medicine, National Yang-Ming University, Taipei, Taiwan; 3 Department of Medical Education, and Pediatrics, Taipei Veterans General Hospital, Taipei, Taiwan; 4 Department of Urology, School of Medicine, Shu-Tien Urological Research Center, National Yang-Ming University, Taipei, Taiwan; 5 Division of Male Reproductive Medicine, Department of Urology, Taipei Veterans General Hospital, Taipei, Taiwan; Semmelweis Egyetem, HUNGARY

## Abstract

This study aimed to investigate the effects of anti-tumor necrosis factor (TNF)-α antibody (Ab) on alteration of penile structure in the hyperprolactinemia (hyperPRL) rat model. HyperPRL was induced in 8-week-old male Sprague-Dawley rats by allografting anterior pituitary (AP) glands under the renal capsule (+AP rats). Rats implanted with cerebral cortex (CX) were used as sham control (+CX rats). At 6 weeks post implantation, rats received either a single intra-testicular dose of TNF-α Ab (12.5 μg/kg) or testosterone replacement (2 doses of testosterone enanthate [TE], 3 mg/kg), and they were sacrificed 1 week later. Blood and penile tissue was collected for analysis. Compared to +CX rats, the +AP group had lower serum testosterone concentration and neuronal nitric oxide synthase (nNOS) expression, but exhibited a higher ratio of collagen III/I in the corpus cavernosum. Smooth muscle content exhibited no significant changes. At 1 week post TNF-α Ab injection, the collagen III/I ratio in the +AP group was decreased, and the smooth muscle content and nNOS expression increased significantly. These findings were comparable to those observed in +AP rats receiving TE. Testicular TNF-α suppresses testosterone release, which in turn results in the erectile dysfunction (ED) seen in hyperPRL. Intra-testicular TNF-α Ab treatment is as effective as testosterone supplementation on penile structure normalization in the hyperPRL model.

## Introduction

Prolactin (PRL), a 23 kDa peptide, is secreted from the lactotrophs of the anterior pituitary (AP) gland under the inhibitory control of hypothalamic dopamine. The main functions of PRL in females are inducing and maintaining lactation during the peripartum and postpartum phases. In males, the role of PRL is less significant. However, a PRL deficiency in childhood might interfere with development of the reproductive system [1, 2]. Overproduction and subsequently increased blood PRL level, known as hyperprolactinemia (hyperPRL), may be seen in various physiological states, such as pregnancy, lactation, other pathological conditions (e.g., tumor growth in the pituitary/hypothalamus region), or medications that reduce dopamine levels in the central nervous system (CNS). Men with hyperPRL may experience symptoms, including galactorrhea, hypogonadism, lower libido, infertility, or erectile dysfunction (ED) [3]. Previous studies have investigated the effects of hyperPRL on sexual function. For instance, we found that the penile structure of the hyperPRL rodent model exhibits lower intra-cavernosal pressure in response to cavernosal nerve stimulation or intra-cavernosal administration of vasoactive agents [4]. Rehman and colleagues demonstrated that hyperPRL induced in rats by acute ovine PRL (oPRL) injection abolished penile reflexes, including erections, cups, and flips [5]. In a study of dogs, oPRL infusion into the corpus cavernosum resulted in significant suppression of intra-cavernous pressure [6]. Hence, acute hyperPRL appears to have a direct inhibitory effect on cavernous smooth muscle contraction.

In clinical practice, antipsychotics and antidepressants used to treat psychiatric diseases, behavioral disorders, or depression usually result in lowering CNS dopamine levels and thus hyperPRL [7]. The occurrence of sexual dysfunction has been commonly reported in patients receiving antipsychotics or antidepressants [8, 9], and these patients are more prone to hypogonadism [10]. Moreover, for ED patients receiving antipsychotic or antidepressant medications, treatment with phosphodiesterase 5 inhibitors, such as sildenafil (Viagra), are less effective [11, 12]. Currently, the major medical treatment for hyperPRL is administration of dopamine-agonists; however, this therapy is not appropriate for patients with underlying psychiatric or psychotic disorders, because suppressing dopamine release is critical for managing their underlying problems. Therefore, other treatment strategies are mandatory to improve such circumstances.

Studies have shown that TNF-α can affect erectile function by reducing neuronal nitric oxide synthase (nNOS) expression, promoting inflammation and fibrosis [13]. In addition to the finding of lower intra-cavernosal pressure, our previous studies of the hyperPRL rat model have demonstrated that, compared to normal male rats, significantly more TNF-α is secreted by the testicular interstitial macrophages and is associated with suppression of gonadotropin-induced testosterone release by Leydig cells [14–16]. TNF-α secretion by isolated testicular interstitial macrophages in prolactin-conditioned medium was significantly increased [14]. We also found that intra-testicular administration of anti-TNF-α antibody can reverse hyperPRL-related hypogonadism [16]. Carneiro et al. reported that the corpora cavernosa of TNF-α knockout mice exhibit increased nitric oxide (NO)-dependent relaxation associated with increased expression of penile nNOS and endothelial NO synthase (eNOS) [17]. They also showed that, in normal mice, TNF-α infusion induces decreased nonadrenergic noncholinergic-mediated relaxation, decreased eNOS and nNOS expression, and increased corpora cavernosa responses to adrenergic nerve stimulation [18]. Men with TNF-α-related ankylosing spondylitis reported significant improvement in erectile function after three months of TNF-α blocker therapy [19].

Good penile erection determined by pressure maintenance in the cavernosal sinuses and it relies on competent functions of endothelium, collagen, smooth muscle and elastic fibers. Changes of the connective tissue and smooth muscle in rabbits with erectile dysfunction can be quantitatively assessed by collagen III/I and collagen/smooth muscle ratio [20]. Based on our previous findings and those of others, we hypothesized that testicular TNF-α may play a detrimental role in erectile function in rats with hyperPRL. The purpose of this study was to investigate the effects of testicular interstitial TNF-α on penile structure alteration and molecular regulation in the hyperPRL rat and then elucidate the mechanism of TNF-α action on erectile function.

## Materials and methods

### Animals

Male Sprague-Dawley rats (8 weeks old) weighing 250 to 300 g were purchased from the Animal Center of National Yang-Ming University and housed at 22 ± 1°C, with a light cycle between 08:00h and 20:00h. Food and water were provided *ad libitum*. All animal experiments were approved by the Institutional Animal Care and Use Committee of National Yang-Ming University. Approval number IACUC 2012–074, IACUC 2013–143.

### Induction of hyperPRL

HyperPRL was induced by allografting AP glands under the left renal capsule as previously described [16]. Briefly, rats were anesthetized with an intra-muscular injection of Zoletil (40 mg/kg), and an incision was made in the left flank region to expose the kidney. At the renal equator, a small incision was made in the renal capsule and the subcapsular space expanded to allow accommodation of two AP glands [14]. Grafted AP glands were pushed cephalad against the upper pole of the renal capsule, and the kidney was then placed back into the peritoneal cavity. For sham control animals, the same procedure was conducted, except that the graft consisted of an equivalent amount of cerebral cortex (CX). Stable hyperPRL status was achieved after 6 weeks post AP implantation. AP allografts of those rats whose serum PRL concentrations were increased by at least 50% above mean sham control concentration were considered successful, and those rats were retained for further experiments. These AP-grafted rats exhibited hyperPRL without changes in the other pituitary hormones, and the CX-grafted rats showed the same hormone profile as that of untreated control rats [14]. Subsequent experiments were performed starting at the 7th week. Upon completion of the experiments, rats were anesthetized, decapitated, and the sera and penises collected for future hormone measurement and histomorphometric analysis, respectively. We used anesthesia to reduce the suffering and distress of animals before any process that is potentially stressful or painful. The rats were continuously examined and monitored during the experiment, and there were no unexpected deaths in this study.

### Experimental design and treatment

Rats were randomly divided into the following six treatment groups (*n* = 8 per group): (1) rats implanted with CX (+CX, sham control); (2) rats implanted with AP (+AP); (3) and (4) +CX and +AP rats, respectively, with saline-dissolved anti-TNF-α antibody (Ab,12.5 μg/kg, R&D Systems, Minneapolis, MN, USA) injected 1× into the left testis at 1 week before experiment; and (5) and (6) +CX and +AP rats, respectively, with olive oil-dissolved testosterone enanthate (TE, 3 mg/kg, Fuji Pharma, Tokyo, Japan) injected 2× intra-muscularly within 1 week, 3 days apart.

### Blood collection

Blood was collected at decapitation, allowed to settle and coagulate for 20 min, and centrifuged at 1,600 *g* for 20 min. Serum was then transferred to a new tube. All serum samples were stored at −20°C until analysis.

### Prolactin analysis

Serum prolactin concentration was analyzed with a PRL ELISA kit (Bertin Pharma, Montigny Le Bretonneux, France). According to the manufacturer, the assay detection range was 0.39–50 ng/ml and the detection limit was 0.2 ng/ml.

### Testosterone analysis

Serum (0.1 ml) was mixed with 0.4 ml diethyl ether, shaken for 1 h, and quickly frozen in an ethanol/dry ice mixture. The organic phase (supernatant) was collected, dried, and reconstituted in a buffer solution (0.1% gelatin in PBS, pH 7.5) before measuring testosterone concentration by radioimmunoassay [21]. Sensitivity of the anti-testosterone serum (no. W8) was 2 pg per assay tube [21].

### TNF-α measurement

Serum TNF-α was measured with the Rat TNF-α/TNFSF1A ELISA kit (R&D Systems). According to the manufacturer, the assay detection range was 12.5–800 pg/ml. The detection limit was typically less than 5 pg/ml.

### Histomorphometric analysis

#### Tissue section preparation

Penile tissues were fixed with 4% paraformaldehyde in PBS (pH 7.4) for 24 h. After PBS washing, tissues were dehydrated and embedded in paraffin at 60°C. Transverse tissue sections at 4-μm thickness were obtained.

#### Collagen I and III distribution

Picrosirius red stain (Polyscience Inc., Warrington, PA, USA) was used to distinguish collagen I (red/orange) from collagen III (green) under polarized microscopy. Eight randomly chosen cavernosal tissue areas were captured per slide, and areas of collagen I and III were measured with the Image J version 1.45s Threshold Color plugin (National Institutes of Health, Bethesda, MD, USA). Data was expressed as the ratio of collagen III/I (%).

#### Smooth muscle content

Masson’s trichrome stain was used to differentiate areas of smooth muscle and collagen in the corpus cavernosum. Eight randomly chosen smooth muscle areas were quantified by the Image J Threshold Color plugin. The entire corpus cavernosum area was manually delineated, and quantitative results were expressed as percentage of smooth muscle within the corpus cavernosum.

#### nNOS expression

Penile dorsal nerve nNOS expression was analyzed by immunofluorescence microscopy using primary rabbit nNOS polyclonal Ab (1:100 dilution, Cell Signaling Technology, Inc., Danvers, MA, USA) and secondary antibody conjugated with rhodamine. Anti-β3-tubulin Ab (1:100 dilution, Novus Biologicals, Littleton, CO, USA) and secondary Ab conjugated with fluorescein were used to detect nerve fibers. Eight randomly chosen areas were quantified and the entire dorsal nerve area manually delineated as described above. Quantitative results were expressed as percentage of nNOS within the dorsal nerve area.

#### TNF-α and macrophage expression

To confirm whether macrophages or TNF-α are present in the penis in our animal model, we used immunohistochemistry to detect expression of TNF-α and the macrophage marker, ED1, in penis sections. Primary anti-TNF-α Ab (1:100 dilution) was obtained from R&D Systems. Anti-ED1 primary Ab (1:100 dilution) was obtained from Hycult Biotech (Uden, Netherlands). Splenic tissue was used as a negative (skipped the primary antibody step) and positive control.

### Statistical analysis

All statistical analyses were performed in the SPSS software package (version 17.0, SPSS Inc., Chicago, IL, USA). Quantitative data were expressed as the mean ± standard error of the mean (SEM). Analysis of variance with Fisher’s least significant difference post hoc test was used for group comparisons. Difference between two means was tested for significance by Student’s *t*-test, with *P*< 0.05 considered as statistically significant.

## Results

### Serum prolactin

**Fig 1:**
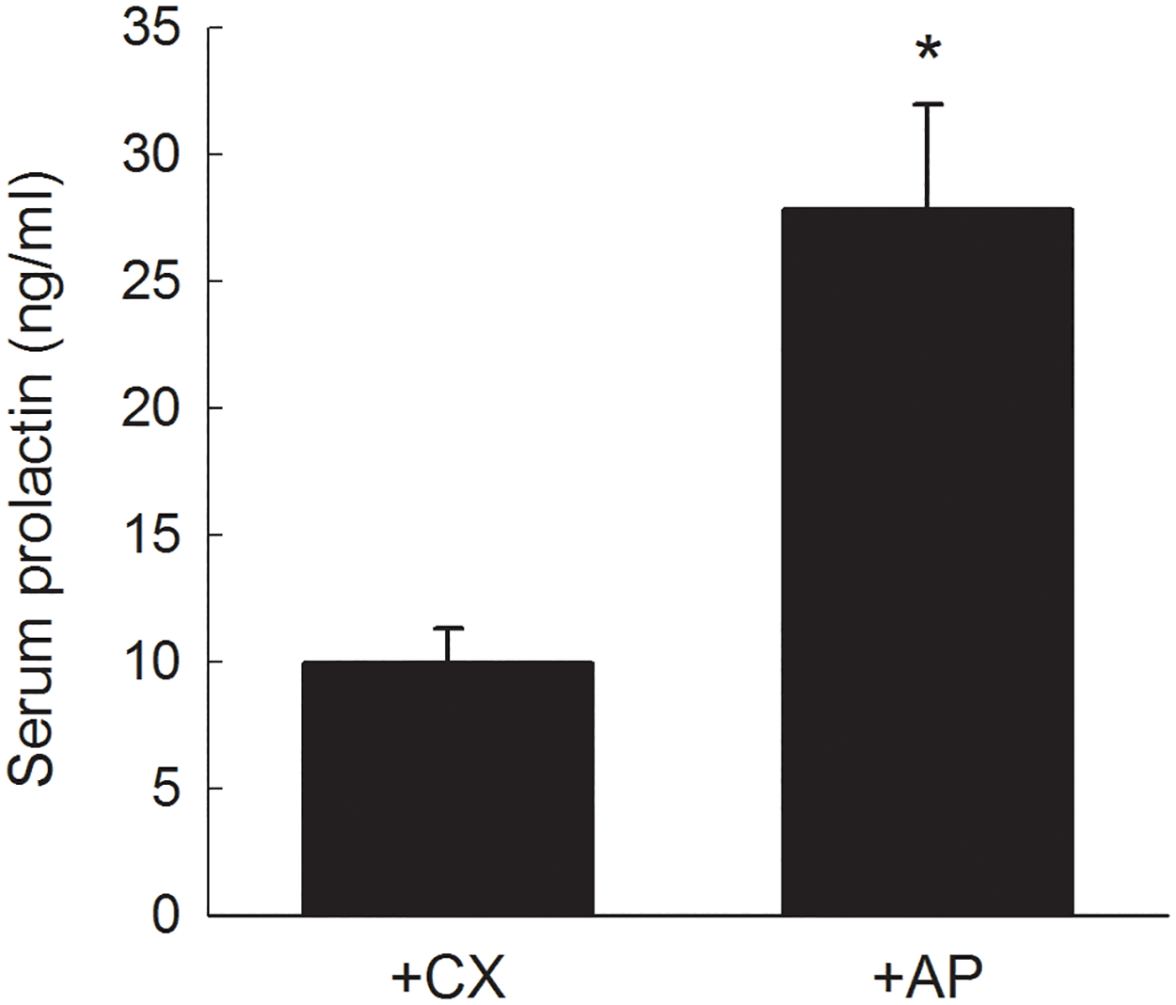
Serum PRL in +AP and +CX rats. Each column represents the mean ± SEM of 8 rats. *P< 0.05 vs. +CX group.

Serum PRL levels were examined to confirm the success of the hyperPRL rat model. As shown in Fig 1, mean serum PRL level in the +AP group was approximately 3-fold higher than that in the +CX group (27.8 ± 4.1 ng/ml vs. 9.9 ± 1.4 ng/ml, *P*<0.05).

### Histomorphometric analysis

#### Collagen fibers

**Fig 2:**
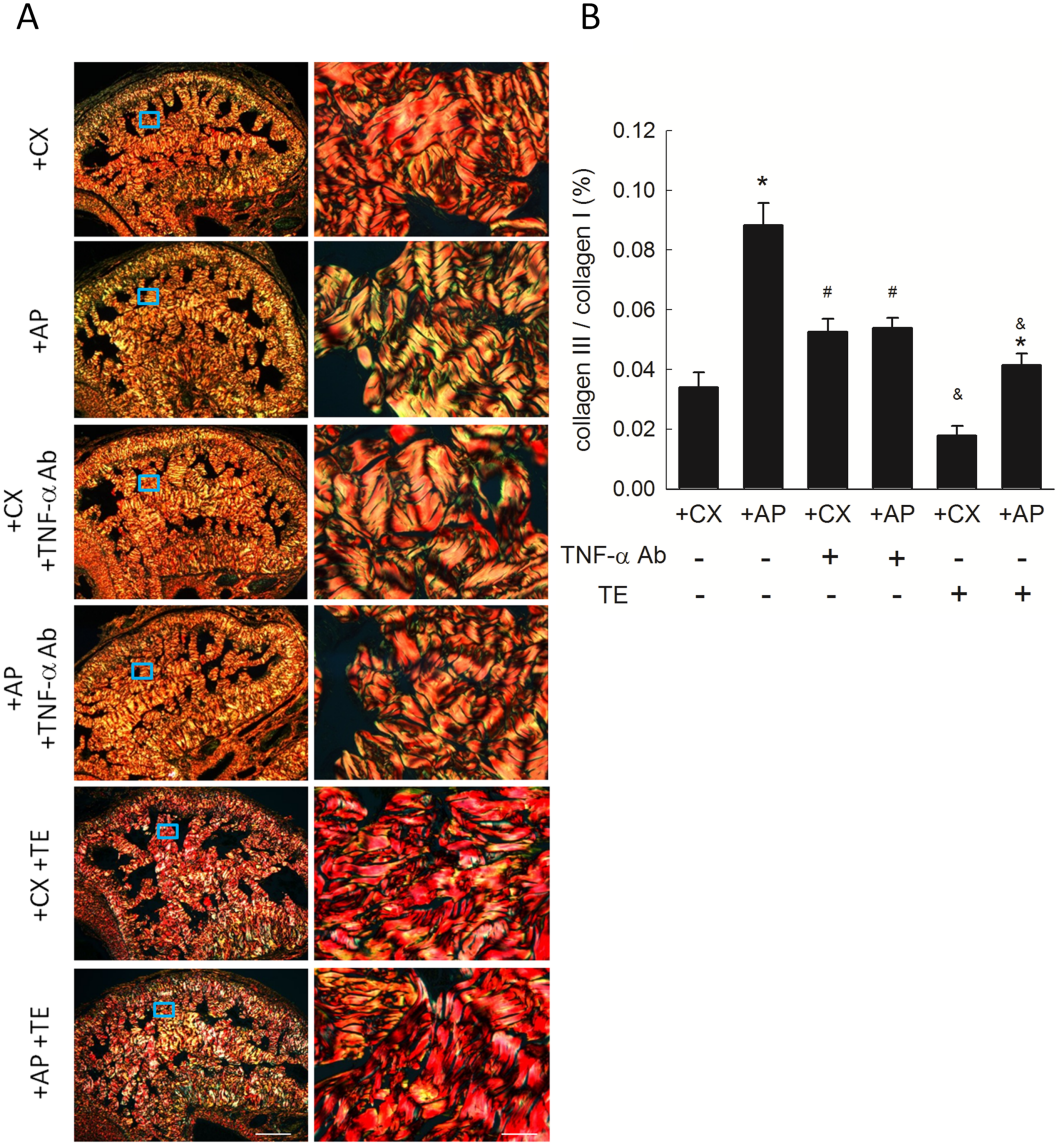
Collagen distribution in rat corpus cavernosum. (A) Representative photomicrographs of picrosirius red-stained corpus cavernosum showing collagen I (red/orange) and collagen III (green). Each right photomicrograph is the magnification of the box in the corresponding left photomicrograph. Scale bar represents 500 μm (left) and 50μm (right). (B) Collagen III/I ratio. Each column represents the mean ± SEM of representative photomicrograph taken from 8 rats. *P<0.05, +AP vs. untreated +CX. ^#^*P*<0.05, TNF-α Ab-treated vs. respective untreated groups. ^&^*P*<0.05, TE-treated vs. respective untreated groups.

The differential distribution of collagen I and III in the corpus cavernosum is shown in Fig 2A. Mean collagen III/I ratio (Fig 2B) in the +AP group was greater than that in the +CX group (0.088% vs. 0.034%, *P*<0.05). One week after TNF-α Ab or TE injection, the collagen III/I ratio in +AP rats was significantly decreased compared to that in the untreated +AP group. Compared to that in the untreated +CX group, collagen III in the +CX group receiving TE was also significantly decreased. These findings suggested that intra-testicular TNF-α Ab injection and intra-muscular TE injection has similar effects on penile tissue.

#### Smooth muscle content

**Fig 3:**
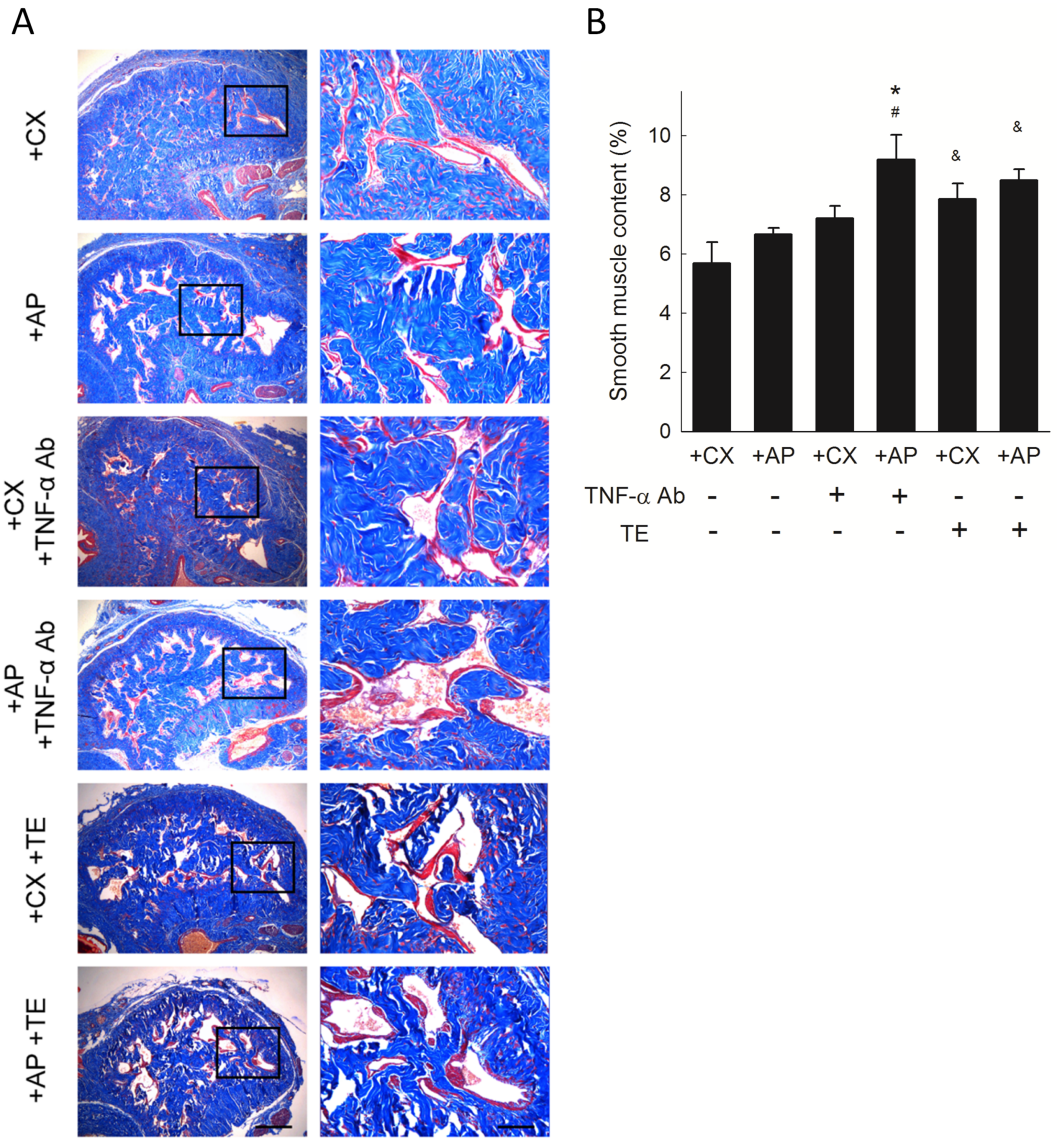
Smooth muscle content in rat corpus cavernosum. (A) Representative photomicrographs of Masson’s trichrome-stained corpus cavernosum showing smooth muscle (red) and collagen (blue). Each right photomicrograph is the magnification of the box in the corresponding left photomicrograph. Scale bar represents 500 μm (left) and 100 μm (right). (B) Smooth muscle content as percentage of corpus cavernosum. Each column represents the mean ± SEM of 8 rats. *P<0.05, TNF-α Ab-treated +AP vs. untreated +CX, ^#^*P*<0.05, TNF-α Ab-treated +AP group vs. untreated +AP group. ^&^*P*<0.05, TE-treated vs. respective untreated groups.

The distribution of smooth muscle and collagen in the corpus cavernosum is shown in Fig 3A. No significant difference in corpus cavernosum smooth muscle content was observed between untreated +AP and +CX rats (Fig 3B). After TNF-α Ab treatment, the +AP group showed a significant increase in smooth muscle content compared to that in untreated +AP and TNF-α Ab treated +CX rats. TE-treated +CX and +AP groups both showed significantly increased smooth muscle content compared to their respective baseline values (Fig 3B).

#### nNOS expression in penile dorsal nerve

**Fig 4:**
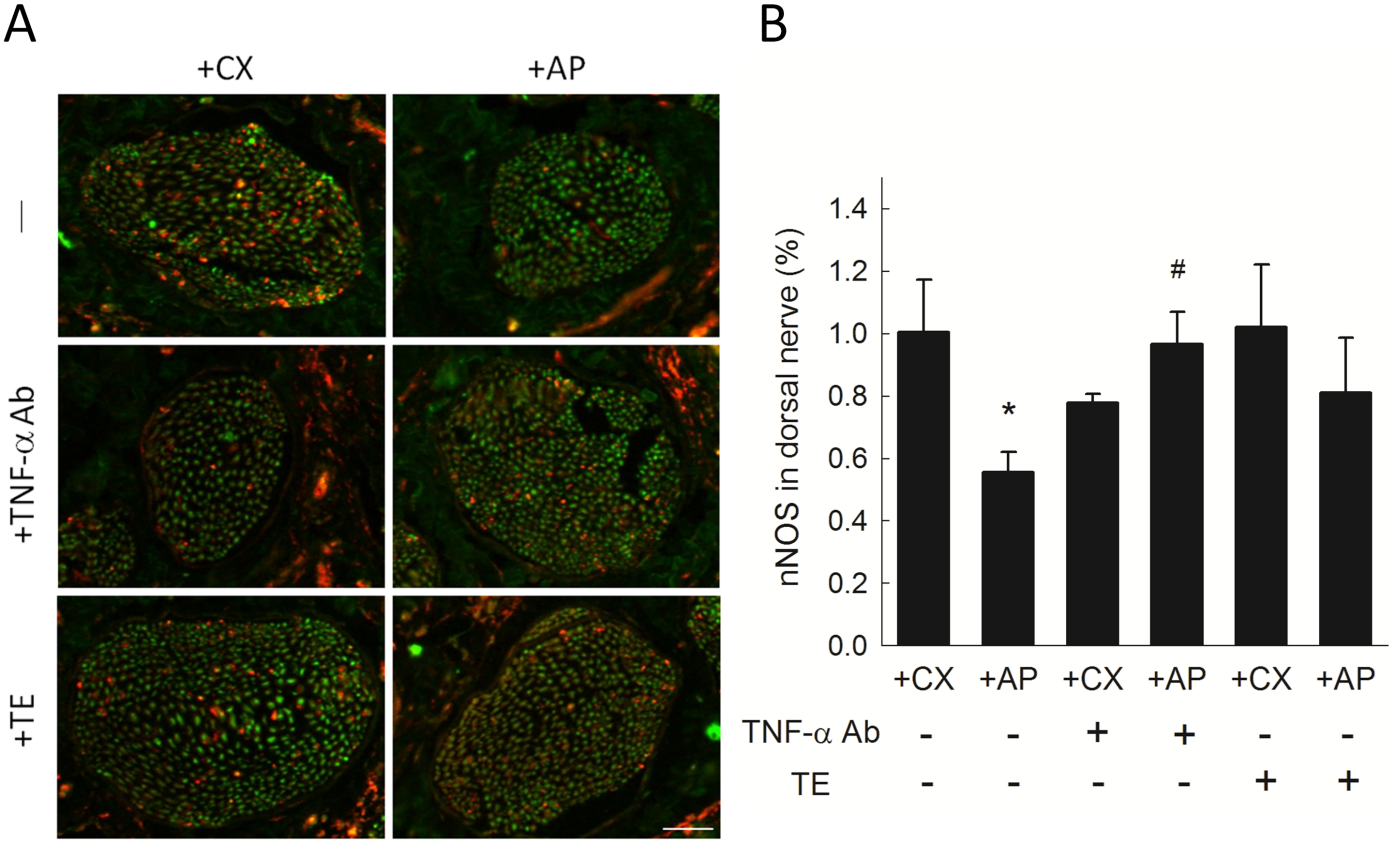
nNOS expression in rat penile dorsal nerve. (A) Representative photomicrographs of immunofluorescent-stained dorsal nerve areas showing nNOS-positive (red) and negative (green) nerve fibers. Scale bar represents 50 μm. (B) nNOS expression as percentage of dorsal nerve area. Each column represents the mean ± SEM of 8 rats. *P<0.05 vs. untreated +CX group. ^#^*P*<0.05, TNF-α Ab-treated vs. untreated +AP group.

Dorsal nerve nNOS expression is shown in Fig 4A. The +AP rats exhibited fewer nNOS-positive areas compared to those in +CX rats. After TNF-α Ab treatment, nNOS expression in the +AP group increased significantly up to the levels of +CX control (Fig 4B). Although TE treatment demonstrated trend of increased nNOS expression, but it did not reach statistically significant difference.

#### Penile TNF-α and ED1 expression

In penile sections of our animal model, only trace amounts of TNF-α could be detected in the corpora cavernosa of +AP and +CX rats, and no difference could be found between them (Figure in S1 Fig). However, in the corpora cavernosa of either the +AP or the +CX group, there was no ED1 immunostaining observed (Figure in S2 Fig). Compared to the positive control shown in spleen tissue, the finding in the penis supports that macrophages are not involved in the penile tissue in hyperPRL status.

### Serum testosterone and TNF-α

**Fig 5:**
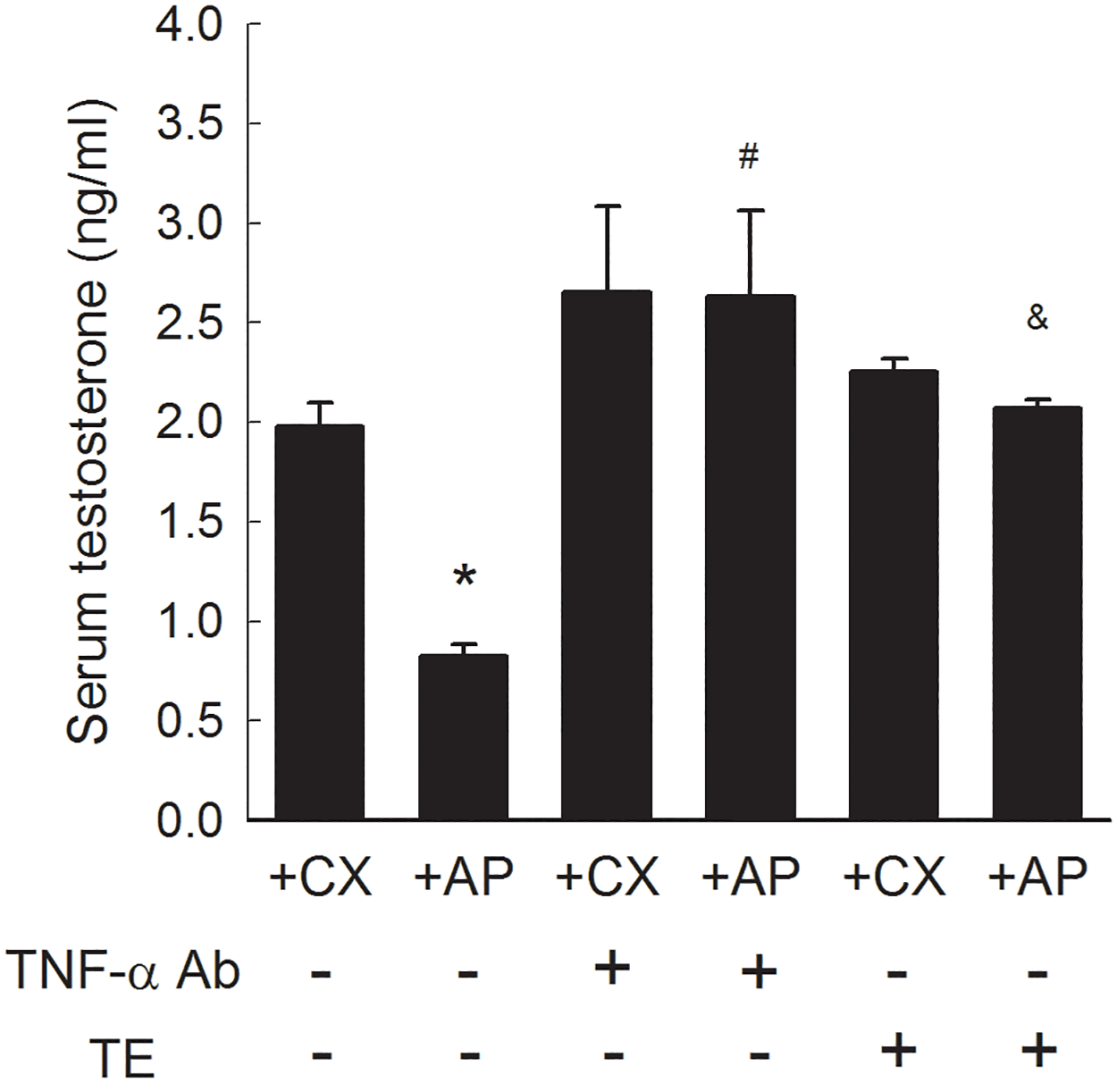
Serum testosterone concentration in response to TNF-α Ab or TE treatment. Each column represents the mean ± SEM of 8 rats. *P<0.05 vs. untreated +CX group. ^#^*P*< 0.05, TNF-α Ab-treated vs. untreated +AP group. ^&^*P*< 0.05, TE-treated vs. untreated +AP group.

Serum testosterone levels were examined in rats at sacrifice. Serum testosterone concentration in +AP rats was lower than that in +CX rats (Fig 5). The +AP rats exhibited increased serum testosterone when they were pre-treated with TNF-α Ab or TE, (*P*<0.05 vs. untreated +AP group). Regarding the serum TNF-α levels, there was no difference detected between +AP and +CX rats (Figure in S3 Fig).

## Discussion

This study investigated the role of testicular interstitial TNF-α in hyperPRL-related ED. The hyperPRL rat model exhibited increased penile collagen III/I ratio (Fig 2) and decreased nNOS expression (Fig 4). Although no significant change in smooth muscle content (Fig 3) and serum TNF-α (Figure in S3 Fig) was observed in the +AP rats, serum testosterone was decreased in these animals (Fig 5). This latter finding agrees with the results of our previous studies, which showed that supraphysiological PRL may suppress hCG-induced testosterone secretion via the inhibitory effects of TNF-α [15, 16]. The changes in penile structure that we did observe appear to be primarily testosterone dependent, as we could not detect TNF-α (Figure in S1 Fig) or macrophages (Figure in S2 Fig) in the corpora cavernosa or detect TNF-α in the sera of these rats (Figure in S3 Fig). Therefore, ED in our hyperPRL model is not likely due to the direct TNF-α effects on the penis.

However, when the +AP rats received a single intra-testicular injection of TNF-α Ab, we observed a rapid decrease in collagen III/I ratio (Fig 2) and an increase in smooth muscle (Fig 3), nNOS expression (Fig 4), and serum testosterone (Fig 5). These findings confirmed our hypothesis that testicular TNF-α may play a role in impairment of erectile function in the hyperPRL model, and they suggested that the mechanism of testicular TNF-α action in this regard is suppression of testosterone secretion. We therefore attempted to elucidate this hypothetical mechanism by treating +AP rats with TE and comparing the resulting penile structure changes to those observed after TNF-α Ab treatment. We found that TE treatment normalized the collagen distribution (Fig 2) and increased the smooth muscle amount (Fig 3) to a similar extent as after the TNF-α Ab treatment. Hence, these results support our hypothesis that testicular TNF-α exhibits its inhibitory effect on erectile function in hyperPRL.

Testosterone is an important inducer of stem cell differentiation [22], and endogenous muscle-derived stem cells have been detected in rat corpora cavernosa [23]. *In vitro* stem cell treatments can improve muscle content and decrease fibrosis in the corpora cavernosa [24]. Moreover, testosterone replacement therapy can increase the number of circulating endothelial progenitor cells in men with late onset hypogonadism [25]. Therefore, we believe that mesenchymal stem cells in the penis are rapidly recruited in response to sufficient testosterone supply. Studies on dynamic changes in stem cells in response to testosterone are currently underway.

Although testosterone replacement therapy is able to restore NOS-containing nerve fibers in corpora cavernosa of castrated rats’ model [26], in our study the expression of nNOS in penis of +AP rats did not increase significantly after TE treatment (Fig 4). This may imply that the recovery of nNOS expression after intra-testicular TNF-α Ab treatment in the +AP rats might be due to effects other than restoration of testosterone.

Patients presented with ED after receiving antipsychotic or antidepressant treatments had poorer response to phosphodiesterase type 5 inhibiters (PDE5i) [12]. Since hyperPRL is almost always present alongside the treatments with antipsychotics and antidepressants [7], so hyperPRL is noticed as a cause to impair PDE5i efficacy. In practice, it is not possible to use dopamine agonists in managing hyperPRL in these patients. The results of the present study indicate the potential role of anti-TNF-α Ab to improve the antipsychotic- or antidepressant-induced ED.

Regarding the route of drug administration, intra-testicular way is definitely not practical in clinical setting. Some more less-invasive routes should be developed. Using intra-testicular injection is based on the rationale that the TNF-α in our model is released from the testicular interstitial macrophages [15]. Our previous study has demonstrated that TNF-α Ab given intra-muscularly is as effective in increasing testosterone secretion as given intra-testicularly [16]. Further study is required to confirm the effects of intra-muscularly administered TNF-α Ab on the change of penile structures. Phytochemicals with anti-inflammatory activities in plant diets or pharmaceuticals, like pterostilbene and resveratrol can also serve as treatment to modify high plasma PRL levels [27]. The effects of these oral formula upon the penile structure in the +AP hyperPRL model deserve further investigations.

Our study has some limitations that warrant discussion. First, we examined only morphological changes in penile structure without consideration of physiological or behavioral modifications. Second, we used polyclonal anti-TNF-α Ab, which is not identical to treatment agents used for inflammatory diseases. Additional studies using etanercept or infliximab in the hyperPRL model are therefore needed.

## Conclusion

Our study shows that testicular TNF-α decreases testosterone release and thus remotely induces subsequent penile structure disruption. To our knowledge, this study is the first to suggest a role of TNF-α in hyperPRL-related ED. Anti-TNF-α treatment rapidly recovers testosterone secretion and restores normal penile architecture. These findings imply that anti-TNF-α treatment may lead to beneficial effect on erectile function and that administration of TNF-α Abs may be a promising approach in managing ED in patients with psychotropic-induced hyperPRL.

## Supporting information

S1 Table S1A Table. Serum PRL (ng/ml) in +AP and +CX Rats. S1B Table. Collagen Distribution (%) in Rat Corpus Cavernosum. S1C Table. Smooth Muscle Content (%) in Rat Corpus Cavernosum. S1D Table. nNOS Expression (%) in Rat Penile Dorsal Nerve. S1E Table. Serum Testosterone Concentration (ng/ml) in Response to TNF-α Ab or TE Treatment.
(DOCX)

S1 Fig TNF-α expression in corpus cavernosum. The picture of corpus cavernosum in +CX and +AP rats both showed negative for TNF-α expression. Splenic tissue was used as a negative (skipped the primary antibody step) and positive control. Arrows indicate representative cells showing positive TNF-α. Scale bar = 100 μm.
(TIF)

S2 Fig ED1 expression for detection of macrophages in corpus cavernosum. Brown color represents macrophage by ED1 antibody immunohistochemistry. Macrophage in the blood of the penile dorsal vein was used as positive control. Arrows indicate representative cells showing positive ED-1. Scale bar = 100 μm.
(TIF)

S3 Fig Serum TNF-α in +AP and +CX rats. Each column represents the mean ± SEM of 4 rats.
(TIF)
