# Supplementary material for: Effects of TNF-α on penile structure alteration in rats with hyperprolactinemia
Source: PLoS One. 2017 Aug 1;12(8):e0181952. doi: 10.1371/journal.pone.0181952 (PMC5538640; doi:10.1371/journal.pone.0181952)
Supplement: S1 Table — S1A Table. Serum PRL (ng/ml) in +AP and +CX Rats. S1B Table. Collagen Distribution (%) in Rat Corpus Cavernosum. S1C Table. Smooth Muscle Content (%) in Rat Corpus Cavernosum. S1D Table. nNOS Expression (%) in Rat Penile Dorsal Nerve. S1E Table. Serum Testosterone Concentration (ng/ml) in Response to TNF-α Ab or TE Treatment. (DOCX) [file pone.0181952.s001.docx]

**S1A Table. Serum PRL (ng/ml) in +AP and +CX Rats.**

| No. | CX | AP |
| --- | --- | --- |
| 1 | 6.46 | 22.32 |
| 2 | 5.25 | 25.41 |
| 3 | 7.66 | 18.87 |
| 4 | 15.98 | 37.24 |
| 5 | 11.32 | 25.30 |
| 6 | 7.50 | 12.14 |
| 7 | 10.41 | 49.74 |
| 8 | 14.90 | 31.72 |
|  |  |  |
| MEAN | 9.93 | 27.84 |
| SD | 3.68 | 10.91 |
| SE | 3.94 | 11.67 |
| SEM | 1.39 | 4.12 |
| n | 8 | 8 |

**S1B Table. Collagen Distribution (%) in Rat Corpus Cavernosum.**

|  |  |  | TNF-α Ab | | TE | |
| --- | --- | --- | --- | --- | --- | --- |
| No. | CX | AP | CX | AP | CX | AP |
| 1 | 0.041 | 0.085 | 0.034 | 0.026 | 0.013 | 0.029 |
| 2 | 0.052 | 0.061 | 0.052 | 0.060 | 0.018 | 0.047 |
| 3 | 0.075 | 0.058 | 0.071 | 0.042 | 0.009 | 0.047 |
| 4 | 0.047 | 0.110 | 0.076 | 0.066 | 0.011 | 0.054 |
| 5 | 0.026 | 0.051 | 0.060 | 0.049 | 0.041 | 0.060 |
| 6 | 0.019 | 0.119 | 0.036 | 0.049 | 0.035 | 0.038 |
| 7 | 0.029 | 0.123 | 0.043 | 0.050 | 0.017 | 0.038 |
| 8 | 0.019 | 0.082 | 0.068 | 0.071 | 0.006 | 0.016 |
| 9 | 0.020 | 0.109 | 0.054 | 0.058 | 0.002 | 0.032 |
| 10 | 0.016 | 0.111 | 0.046 | 0.061 | 0.021 | 0.035 |
| 11 | 0.028 | 0.071 | 0.061 | 0.055 | 0.014 | 0.041 |
| 12 | 0.036 | 0.081 | 0.031 | 0.059 | 0.027 | 0.062 |
|  |  |  |  |  |  |  |
| MEAN | 0.034 | 0.088 | 0.053 | 0.054 | 0.018 | 0.041 |
| SD | 0.017 | 0.024 | 0.014 | 0.011 | 0.011 | 0.013 |
| SE | 0.017 | 0.025 | 0.015 | 0.012 | 0.012 | 0.013 |
| SEM | 0.005 | 0.007 | 0.004 | 0.003 | 0.003 | 0.004 |
| n | 12 | 12 | 12 | 12 | 12 | 12 |

**S1C Table. Smooth Muscle Content (%) in Rat Corpus Cavernosum.**

|  |  |  | TNF-α Ab | | TE | |
| --- | --- | --- | --- | --- | --- | --- |
| n | CX | AP | CX | AP | CX | AP |
| 1 | 5.37 | 6.83 | 7.68 | 7.28 | 10.29 | 9.71 |
| 2 | 5.83 | 7.75 | 8.56 | 13.64 | 8.35 | 8.70 |
| 3 | 3.56 | 6.20 | 5.87 | 7.31 | 6.29 | 8.75 |
| 4 | 3.62 | 7.20 | 9.27 | 6.58 | 6.08 | 10.19 |
| 5 | 6.16 | 6.25 | 6.49 | 10.57 | 8.04 | 7.50 |
| 6 | 6.24 | 7.07 | 6.89 | 9.81 | 7.68 | 8.22 |
| 7 | 9.91 | 5.88 | 6.47 | 10.30 | 9.29 | 7.64 |
| 8 | 4.81 | 6.11 | 6.41 | 8.10 | 6.89 | 7.28 |
|  |  |  |  |  |  |  |
| MEAN | 5.69 | 6.66 | 7.20 | 9.20 | 7.87 | 8.50 |
| SD | 1.87 | 0.61 | 1.11 | 2.19 | 1.36 | 0.98 |
| SE | 2.00 | 0.65 | 1.19 | 2.35 | 1.46 | 1.05 |
| SEM | 0.71 | 0.23 | 0.42 | 0.83 | 0.51 | 0.37 |
| n | 8 | 8 | 8 | 8 | 8 | 8 |

**S1D Table. nNOS Expression (%) in Rat Penile Dorsal Nerve.**

|  |  |  | TNF-α Ab | | TE | |
| --- | --- | --- | --- | --- | --- | --- |
| n | CX | AP | CX | AP | CX | AP |
| 1 | 2.07 | 0.56 | 0.88 | 1.21 | 1.02 | 0.30 |
| 2 | 0.77 | 0.46 | 0.65 | 0.77 | 2.15 | 0.83 |
| 3 | 1.24 | 0.44 | 0.84 | 0.91 | 0.83 | 1.89 |
| 4 | 0.62 | 0.81 | 0.72 | 0.86 | 0.45 | 0.45 |
| 5 | 0.89 | 0.54 | 0.72 | 0.69 | 0.32 | 0.81 |
| 6 | 0.67 | 0.25 | 0.82 | 1.57 | 1.36 | 0.62 |
| 7 | 0.97 | 0.61 | 0.75 | 0.76 | 1.01 | 1.04 |
| 8 | 0.81 | 0.78 | 0.85 | 0.97 | 1.03 | 0.55 |
|  |  |  |  |  |  |  |
| MEAN | 1.01 | 0.56 | 0.78 | 0.97 | 1.02 | 0.81 |
| SD | 0.44 | 0.17 | 0.08 | 0.27 | 0.53 | 0.46 |
| SE | 0.47 | 0.18 | 0.08 | 0.29 | 0.57 | 0.49 |
| SEM | 0.17 | 0.06 | 0.03 | 0.10 | 0.20 | 0.17 |
| n | 8 | 8 | 8 | 8 | 8 | 8 |

**S1E Table. Serum Testosterone Concentration (ng/ml) in Response to TNF-α Ab or TE Treatment.**

|  |  |  | TNF-α Ab | | TE | |
| --- | --- | --- | --- | --- | --- | --- |
| n | CX | AP | CX | AP | CX | AP |
| 1 | 1.81 | 0.87 | 3.14 | 3.60 | 2.49 | 2.16 |
| 2 | 1.52 | 0.92 | 1.47 | 1.83 | 2.49 | 2.29 |
| 3 | 1.92 | 0.86 | 2.53 | 2.63 | 2.14 | 1.94 |
| 4 | 1.94 | 0.53 | 0.73 | 0.65 | 1.90 | 1.90 |
| 5 | 1.90 | 0.65 | 4.91 | 4.86 | 2.26 | 2.06 |
| 6 | 1.92 | 0.80 | 2.76 | 2.22 | 2.22 | 2.00 |
| 7 | 2.14 | 0.97 | 2.65 | 2.61 | 2.28 | 2.09 |
| 8 | 2.70 | 1.01 | 3.03 | 2.65 | 2.24 | 2.13 |
|  |  |  |  |  |  |  |
| MEAN | 1.98 | 0.83 | 2.65 | 2.63 | 2.25 | 2.07 |
| SD | 0.32 | 0.15 | 1.15 | 1.15 | 0.18 | 0.12 |
| SE | 0.34 | 0.16 | 1.23 | 1.23 | 0.19 | 0.13 |
| SEM | 0.12 | 0.06 | 0.44 | 0.44 | 0.07 | 0.04 |
| n | 8 | 8 | 8 | 8 | 8 | 8 |

**S1 Fig. TNF-α** **Expression in Corpus Cavernosum.**


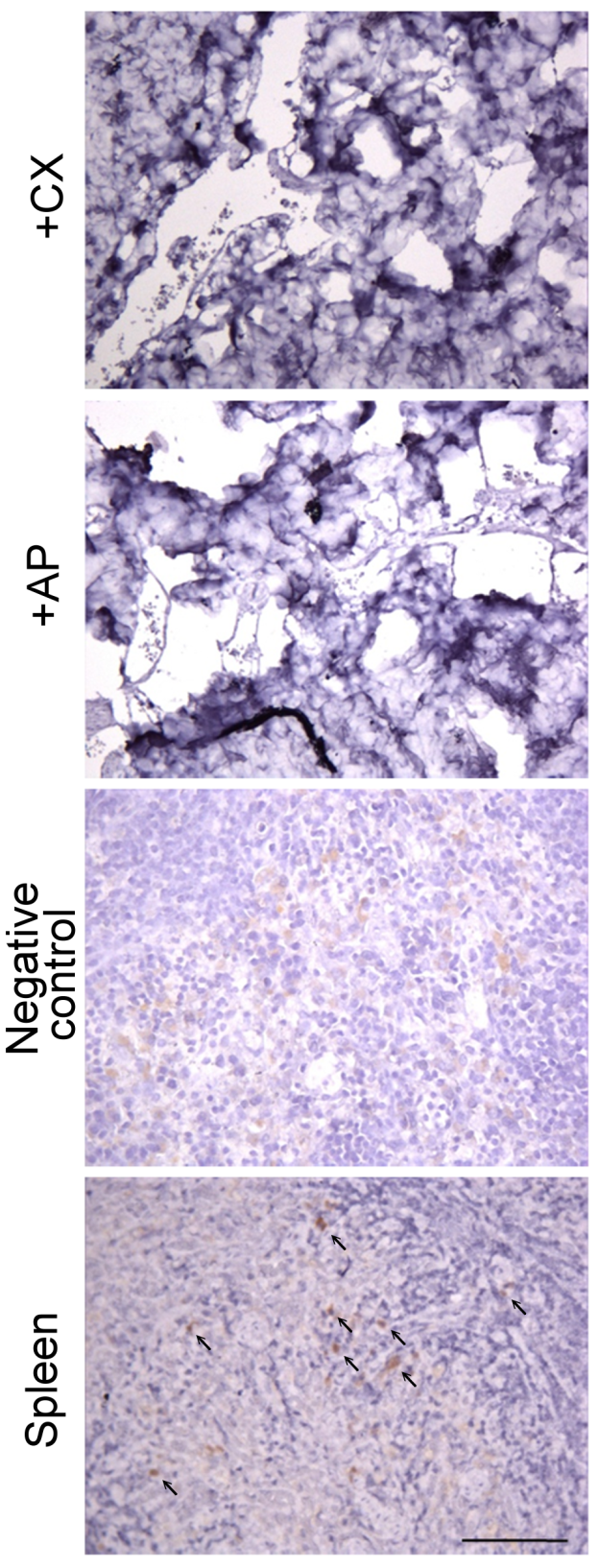


**S2 Fig. ED1 Expression for Detection of Macrophages in Corpus Cavernosum.**


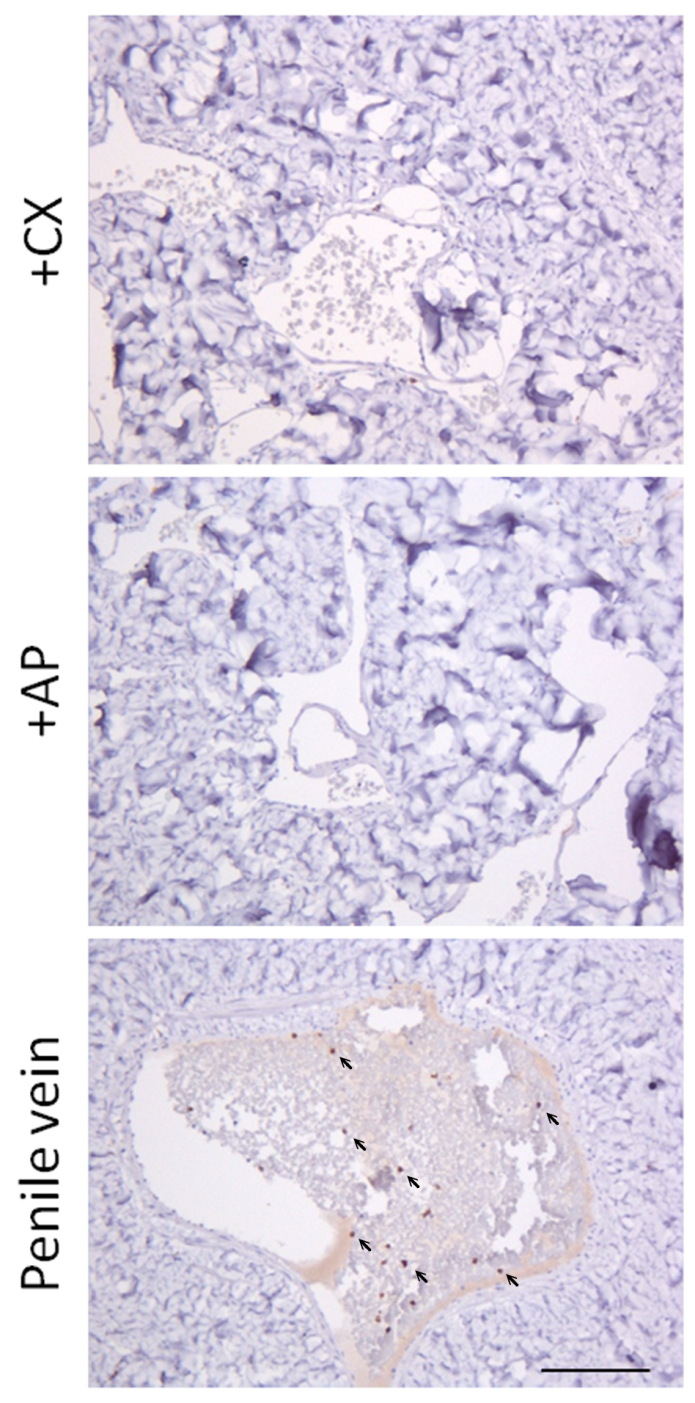


**S3 Fig. Serum TNF-α in +AP and +CX Rats.**





**S3 Fig. Serum TNF-α in +AP and +CX Rats.** Each column represents the mean ± SEM of 4 rats.
