# Supplementary figures and images for: Effects of TNF-α on penile structure alteration in rats with hyperprolactinemia
Source: PLoS One. 2017 Aug 1;12(8):e0181952. doi: 10.1371/journal.pone.0181952 (PMC5538640; doi:10.1371/journal.pone.0181952)

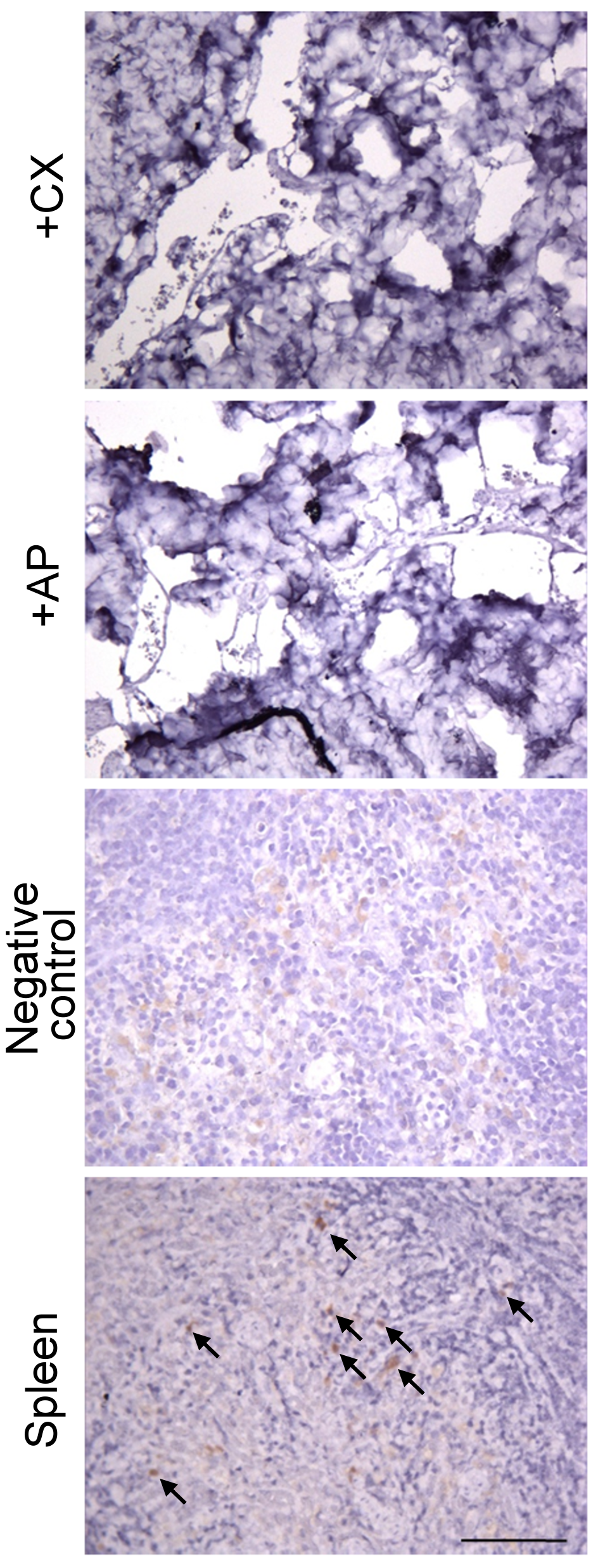

Supplement: S1 Fig — The picture of corpus cavernosum in +CX and +AP rats both showed negative for TNF-α expression. Splenic tissue was used as a negative (skipped the primary antibody step) and positive control. Arrows indicate representative cells showing positive TNF-α. Scale bar = 100 μm. (TIF) [file pone.0181952.s002.tif]

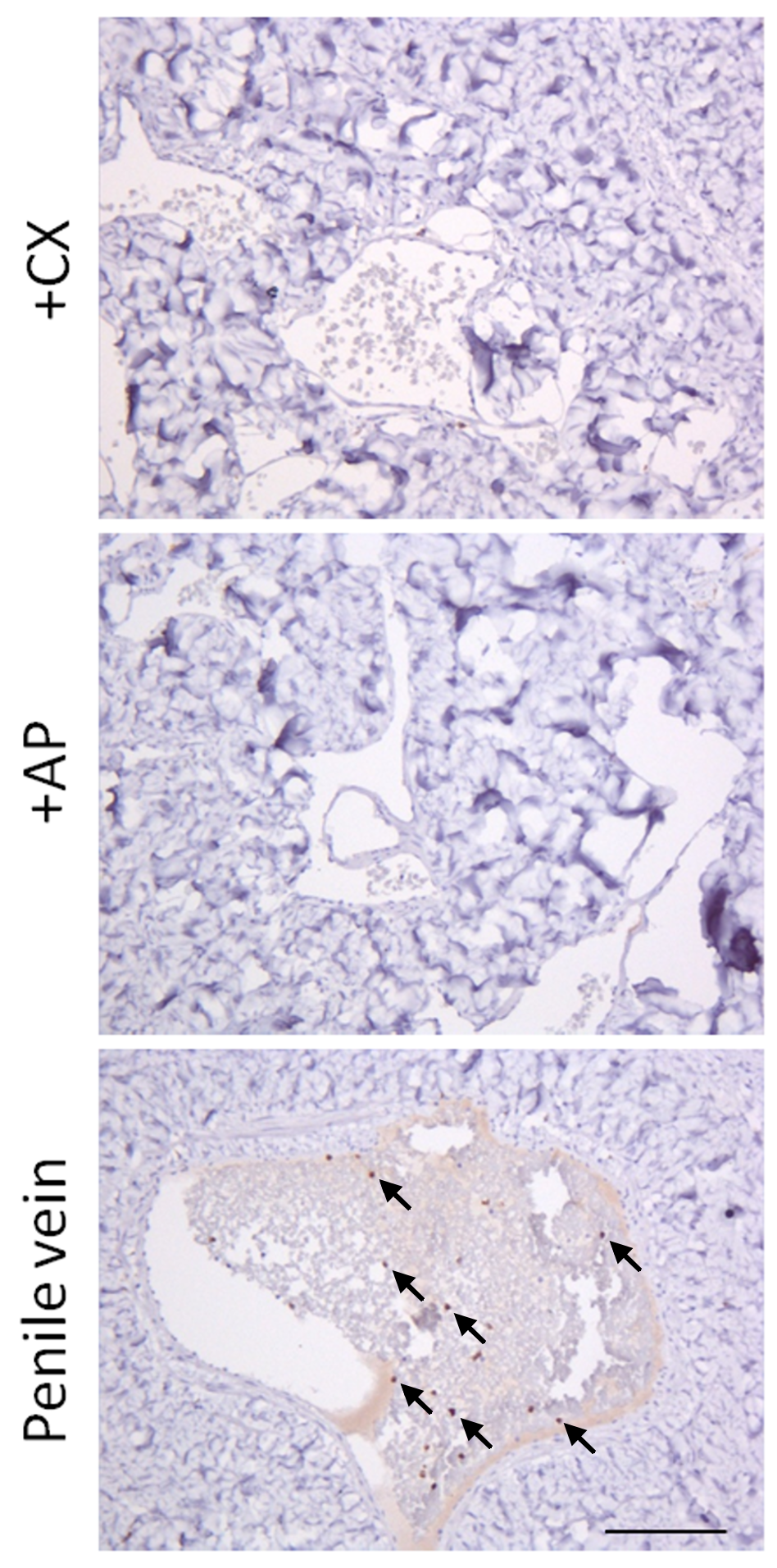

Supplement: S2 Fig — Brown color represents macrophage by ED1 antibody immunohistochemistry. Macrophage in the blood of the penile dorsal vein was used as positive control. Arrows indicate representative cells showing positive ED-1. Scale bar = 100 μm. (TIF) [file pone.0181952.s003.tif]

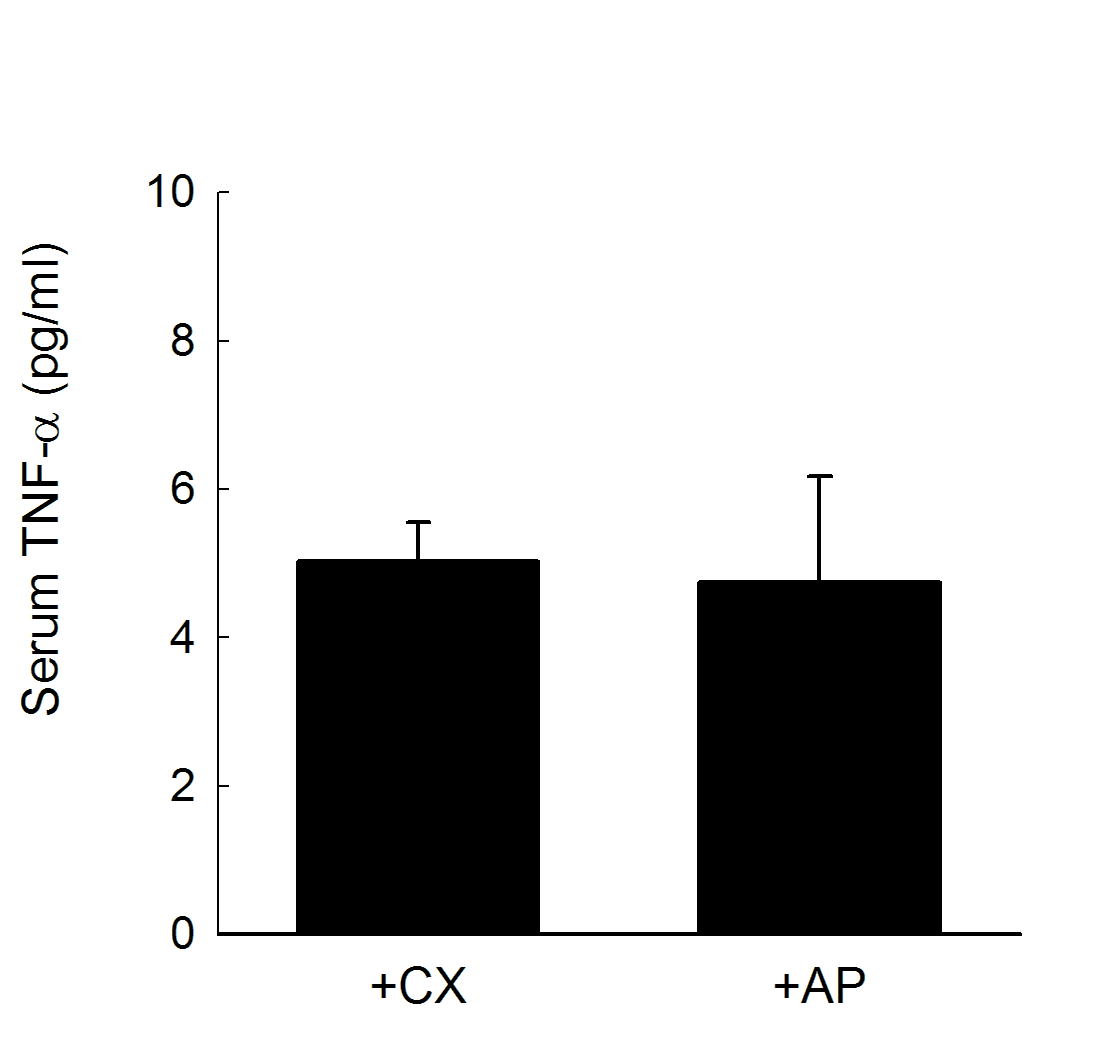

Supplement: S3 Fig — Each column represents the mean ± SEM of 4 rats. (TIF) [file pone.0181952.s004.TIF]
